# CircFAM114A2 Promotes Cisplatin Sensitivity *via* miR-222-3p/P27 and miR-146a-5p/P21 Cascades in Urothelial Carcinoma

**DOI:** 10.3389/fonc.2021.659166

**Published:** 2021-10-14

**Authors:** Jiancheng Lv, Zijian Zhou, Jingzi Wang, Xiao Yang, Hao Yu, Jie Han, Dexiang Feng, Baorui Yuan, Qikai Wu, Pengchao Li, Qiang Lu, Haiwei Yang

**Affiliations:** Department of Urology, First Affiliated Hospital of Nanjing Medical University, Nanjing, China

**Keywords:** circRNA, urothelium cancer, cisplatin, chemotherapy resistance, microRNA

## Abstract

**Introduction:**

Circular RNAs (circRNAs) are non-coding RNAs that have the structure of a covalently closed loop. Increasing data have proven that circRNAs can influence the progression and chemotherapy sensitivity of tumors. Therefore, the underlying function and mechanisms of more circRNAs in progression and chemotherapy resistance are important.

**Methods:**

We conducted RNA sequencing on five pairs of urothelial carcinoma samples and screened for circRNAs. CircFAM114A2 was found to be low expressed in urothelial carcinoma. The functions of circFAM114A2 in urothelial carcinoma were explored by cell cycle assay, IC_50_ determination assay, cell proliferation assay, apoptosis assay, and tumorigenesis assay.

**Results:**

We discovered that the levels of circFAM114A2 were decreased in urothelial carcinoma cell lines and tissues. According to follow-up data, urothelial carcinoma patients with higher circFAM114A2 expression had better survival. Importantly, the levels of circFAM114A2 were associated with the histological grade of urothelial carcinoma. CircFAM114A2 could inhibit cell proliferation and block more urothelial carcinoma cells in the G1 phase and then increase the sensitivity of urothelial carcinoma to cisplatin chemotherapy. Mechanistically, circFAM114A2 directly sponged miR-222-3p/miR-146a-5p and subsequently influenced the expressions of the downstream target genes *P27*/*P21*, which, in turn, inhibited the progression of urothelial carcinoma and increased the sensitivity of cancer cells to cisplatin chemotherapy.

**Conclusion:**

CircFAM114A2 could inhibit progression and promote cisplatin sensitivity in urothelial carcinoma through novel circFAM114A2/miR-222-3p/P27 and circFAM114A2/miR-146a-5p/P21 pathways. CircFAM1142 has therefore great potential as a prognostic biomarker and therapeutic target for urothelial carcinoma.

## Introduction

Urothelial carcinoma has the highest incidence in the urinary system with high morbidity and mortality worldwide (1). It is categorized into muscle invasive bladder cancer (MIBC) (20%–30%) and non-muscle invasive bladder cancer (NMIBC) (70%–80%) (2). Currently, the most common treatments for urothelial carcinoma are surgery, chemotherapy, and radiotherapy. Cisplatin-based adjuvant chemotherapy or neoadjuvant chemotherapy improves the 5-year urothelial carcinoma-specific survival. However, chemotherapy resistance during adjuvant chemotherapy or neoadjuvant chemotherapy makes the treatment more difficult, and the recurrence rate is still very high (3). Therefore, more research on the molecular mechanisms of chemotherapy resistance is needed.

Circular RNA (circRNA) is a new type of non-coding RNA (ncRNA) and consists of a covalent closed-loop assembly minus the 3′ poly A tail and 5′ cap (4). Recently, studies have discovered that circRNAs are intimately related to the chemotherapy resistance of tumors (5). CircRNA Cdr1as improved the sensitivity of bladder cancer to cisplatin through sponging miR-1270 (6). CircBPTF promoted cisplatin chemoresistance in urothelial carcinoma by increasing the expression of RAB27A (7, 8). CircELP3, a hypoxia-induced circRNA, could also promote cisplatin resistance in urothelial carcinoma through acting on cancer stem-like cells (9). Sun et al. also reported that circMCTP2 could inhibit cisplatin resistance in gastric cancer *via* performing as a competitive endogenous RNA (ceRNA) (10). Androgen receptor (AR) has been reported to be involved in the regulation of cisplatin sensitivity (11). AR-mediated circRNA (circFNTA) could inhibited cisplatin sensitivity by the miR-370-3p/FNTA/KRAS pathway (12). Therefore, the underlying functions and mechanisms of more circRNAs in chemotherapy resistance are important.

Cisplatin has always been a major part of the chemotherapy regimen among patients with urothelial carcinoma. Adjuvant chemotherapy and neoadjuvant chemotherapy based on gemcitabine plus cisplatin have become an important treatment scheme in the management of urothelial carcinoma (13). However, the long-term survival of patients is greatly hindered because of chemotherapy resistance to cisplatin (6). According to various studies, cell cycle regulation and cell apoptosis are the theoretical basis of cisplatin action and play important roles in cisplatin chemotherapy resistance (14, 15). *P27* and *P21* are classic tumor suppressors as they play crucial functions in regulating cell cycle transition (16, 17). Ripani et al. reported that *P27* induced cell cycle arrest and increased the sensitivity of colon carcinoma cells to cisplatin (18). Wang et al. reported that *P21* could promote cell cycle arrest and increase the cisplatin sensitivity of urothelial carcinoma (19). In our study, we revealed that circFAM114A2 could sensitize urothelial carcinoma cells to cisplatin *via* enhancing the expressions of *P27* and *P21* by sponging miR-222-3p and miR-146a-5p.

## Materials and Methods

### Clinical Specimens and Cell Lines

Urothelial carcinoma tumor tissues and adjacent normal tissues were acquired from urothelial carcinoma patients who underwent operation at the First Affiliated Hospital of Nanjing Medical University from 2013 to 2017. Utilization of tissues was granted approval by the ethics board at the hospital. Furthermore, a group of 46 urothelial carcinoma patients with clinical–pathological characteristics was monitored. The monitoring period varied between 1 and 62.5 months. Follow-up period was initiated on the day of the operation and lasted until time of disease advancement. The study methodologies conformed to the standards set by the Declaration of Helsinki. The urothelial carcinoma cell lines (SV-HUC, BIU87, TCC, T24, RT4, 5637, 253J, UMUC3, and J82) were acquired through the Type Culture Collection of the Chinese Academy of Sciences (Shanghai, China). Cells were maintained in Dulbecco’s modified Eagle’s medium (DMEM) with 10% fetal bovine serum (FBS) in a 37°C incubator with 5% CO_2_.

### RNA Isolation and qRT-PCR

RNA was extracted through cells and tissues with Trizol as per established guidelines. Relative complementary DNA (cDNA) was produced by utilizing HiScript (Vazyme, Nanjing, China). LightCycler 480 (Roche, Indianapolis, IN, USA) was used for quantitative real-time PCR (qRT-PCR) of the circRNA and microRNAs (miRNAs). U6 and β-actin were selected as controls for circRNA, miRNA, and messenger RNA (mRNA) identification. The CT values of the target RNA were normalized by subtracting the CT value of β-actin. Every experiment was conducted three times and the results were assessed through comparison of the CT values. PCR primers were from Tsingke (Beijing, China) and are listed in **Supplementary Table S1**.

### Protein Extraction and Western Blotting

The cells were lysed using RIPA buffer that contained protease inhibitors (Sigma, St. Louis, MO, USA). Protein was collected and measured using bicinchoninic acid (BCA) assessment. Protein was isolated using 10% SDS-PAGE and moved onto polyvinylidene fluoride (PVDF) membranes. The membrane was incubated with P27 (1:5,000; Abcam, Waltham, MA, USA), P21 (1:5,000, Abcam), or β-actin (1:5,000; Proteintech, Chicago, IL, USA) primary antibody. Thereafter, the membranes were treated with horseradish peroxidase (HRP)-conjugated anti-rabbit secondary antibody (1:1,000; Cell Signaling Technology, Danvers, MA, USA). After conducting washes, the proteins were identified utilizing chemiluminescence and assessed with Image Lab (Bio-Rad, Hercules, CA, USA).

### Transfection

Stable transfection was used in circFAM114A2 relative transfection. Lentivirus constructs containing circFAM114A2 overexpression or knockdown were acquired through OBIO (ObiO Technology Corp., Shanghai, China). T24 and 5637 urothelial carcinoma cells were placed in six-well plates. Cells were then transfected with knockdown lentivirus constructs (si circFAM114A2-1 and si circFAM114A2-2), knockdown negative control (si NC), overexpression lentivirus construct (circFAM114A2), and overexpression negative control (vector) at 50% confluency. The infected cells were treated with puromycin (4 μg/ml) for 2 weeks. The transient transfection method was used in the relative transfection of miRNAs. The microRNA mimics and controls utilized for transfection were from RiboBio (Guangzhou, China). The procedure was conducted with the Lipofectamine 3000 kit (Invitrogen, Carlsbad, CA, USA) as per established guidelines (20).

### Evaluation of Cell Cycle and Apoptosis

To evaluate cell cycle, transfected urothelial carcinoma cells were stained with the cycle test and DNA reagent kit (BD Biosciences, Franklin Lakes, NJ, USA) and quantified through flow cytometry (Becton Dickinson, Franklin Lakes, NJ, USA). Cell proportions in the G0/1, S, and G2/M phases were analyzed using ModFit software (version 5.0). To identify apoptosis, the cells were stained using an annexin V-APC/7AAD apoptosis kit (eBiosciences, San Diego, CA, USA) and assessed by flow cytometry. To investigate the apoptotic rate of urothelial carcinoma cells treated with cisplatin, urothelial carcinoma cells at 70% confluence were treated with 4 µmol/ml cisplatin (TCI, Tokyo, Japan) for 24 h and then collected for follow-up apoptosis testing.

### IC_50_ Determination

The transfected T24 or 5637 urothelial carcinoma cells were gathered and placed in a 96-well plate at 5,000 cells/well. After an overnight incubation, a series of dilute concentrations of cisplatin (128, 64, 50, 40, 32, 16, 8, 4, 2, and 1 µmol/L; TCI, Tokyo, Japan) were added to the transfected cells for 24 h. Thereafter, viability was quantified by the Cell Counting Kit-8 (CCK-8) technique as per established protocol. IC_50_ was generated with the probit regression model (21). Analyses were replicated three times.

### Evaluation of Cell Proliferation and Cloning Formation

To evaluate cell growth, transfected T24 or 5637 urothelial carcinoma cells were placed in 96-well plates with 2,000 or 4,000 cells, respectively, per well. At 24, 48, 72, and 96 h post-seeding, cell viability was calculated through the CCK-8 system as per established guidelines. In summary, 10 μl CCK-8 reagent was placed into each well and the plate was stored at 37°C for 1 h in the dark. Absorbance was quantified (450 nm) using a microplate reader (Tecan, Zurich, Switzerland).

### Biotin-Coupled miRNA Capture

About 2 × 10^6^ urothelial carcinoma cells at 50% confluence were transfected using 50 μM biotinylated miRNA mimics or nonsense control (NC) (RiboBio Guangzhou, China). After 24 h, the cells were collected and washed with phosphate-buffered saline (PBS) twice. Streptavidin magnetic beads (Thermo Fisher, Waltham, MA, USA) were placed in blocking buffer for 2 h and placed into every reaction to extract the biotin-coupled RNA compound. Tubes were placed in incubation while rotating at low speed (10 rpm) for 4 h. The beads were washed by lysis buffer five times. Then, Trizol reagent was utilized to retrieve the RNAs that interacted with the miRNAs. The quantity of circFAM114A2 in bound portions was assessed by qRT-PCR and agarose gel electrophoresis.

### Biotin-Coupled Probe Pull-Down Assay

The biotinylated probe was targeted to bind the junctional region of circFAM114A2. Oligo probe served as the control. Approximately 1 × 10^7^ cells were washed with pre-cooled PBS and then lysed using lysis buffer. The lysed products were placed in incubation using 3 μg biotinylated probes at room temperature for 2 h. Then, the cell lysates were incubated with 50 μl streptavidin magnetic beads for an additional 4 h. The beads were cleaned using lysis buffer five times, and the miRNAs in the pull-down material were isolated utilizing Trizol reagent and assessed with the qRT-PCR assay.

### Agarose Gel Electrophoresis

The agarose gel was made of agarose powder, 1× TAE solution, and nucleic acid dye. About 2 µl DNA ladder was added to 8 µl of the PCR product and loaded to agarose gels. The constant pressure 200 V was set up for 20 min electrophoresis. When electrophoresis was finished, the gel was viewed with an ultraviolet lamp to identify the region of DNA.

### Fluorescence *In Situ* Hybridization

Cy3-labeled probes specific to circFAM114A2 and FAM-labeled probes specific to miR-222-3p/miR-146a-5p were constructed and produced through GenePharma (Shanghai, China). Probe signals were identified through a Fluorescent *In Situ* Hybridization Kit (GenePharma, Shanghai, China) as per established protocol. Pictures were attained on a Zeiss LSM880 NLO confocal microscope system.

### Luciferase Reporter Assay

HEK293 cells were co-transfected with mutant or wild-type 3′-UTR fragments of *P27* or *P21* and miRNA mimics utilizing Lipofectamine 3000 (Invitrogen) as per established instructions. At 24 h post-transfection, the activities of firefly and Renilla luciferase were quantified consistently by utilizing a dual-luciferase reporter assay system (Promega, Madison, WI, USA). Lastly, the proportion of luminescence through luciferase was measured and every experiment was conducted three times.

### Xenografts in Mice

Approximately 1 × 10^7^ cells underwent stable transfection using si circFAM114A2, si NC, circFAM114A2, and vector and were inoculated into the axilla of BALB/C nude mice (4–6 weeks old, 18–22 g, four mice per group) subcutaneously. We divided nude mice inoculated with si circFAM114A2 and si NC transfected cells into four groups, namely, si circFAM114A2+cisplatin group, si NC+cisplatin group, si circFAM114A2+saline group, and si NC+saline group. One week after inoculation, nude mice in the experimental groups were intraperitoneally injected with cisplatin (5 mg/kg) every 3 days, while the control groups were injected with the same volume of saline. Tumor evolution was followed each week, and the width (*W*) and length (*L*) were quantified utilizing calipers. The volume (*V*) of the tumor was measured using the formula *V* = (*W* ^2^ × *L*)/2. After 4 weeks, the mice were sacrificed and the tumor bulk was quantified. Animal experiments were conducted according to ethics guidelines for animal studies and granted approval through the Animal Ethics Board of Nanjing Medical University.

### Immunohistochemistry

Paraffin-embedded tumors from mice were sliced to 4-μm slides. Tissue slides were rehydrated with different grades of ethanol and then placed in sodium citrate buffer (pH 6). Antigen was isolated utilizing a microwave. The slides were dipped in 3% H_2_O_2_ for 10 min and then treated with P27 or P21 antibody at 4°C overnight. After washing, the slides were incubated with the HRP-conjugated antibody and the standard avidin–biotinylated peroxidase complex method. The images were viewed with a microscope. The degree of positivity was assessed by at least two pathologists based on the proportion of positive tumor cells.

### Statistical Analyses

The results were assessed using SPSS version 22.0 and presented as the mean ± standard deviation. The *p*-value was statistically significant when less than 0.05. Two-tailed Student’s *t*-test and one-way ANOVA were conducted to assess variations among the groups. Correlation was assessed through Pearson’s correlation and Spearman’s rank correlation tests. Survival curves were imaged utilizing the Kaplan–Meier method, and variations were assessed through a log-rank test.

## Results

### CircFAM114A2 Was Downregulated in Urothelial Carcinoma and Associated With Cell Cycle Regulation Pathways

**Figure 1:**
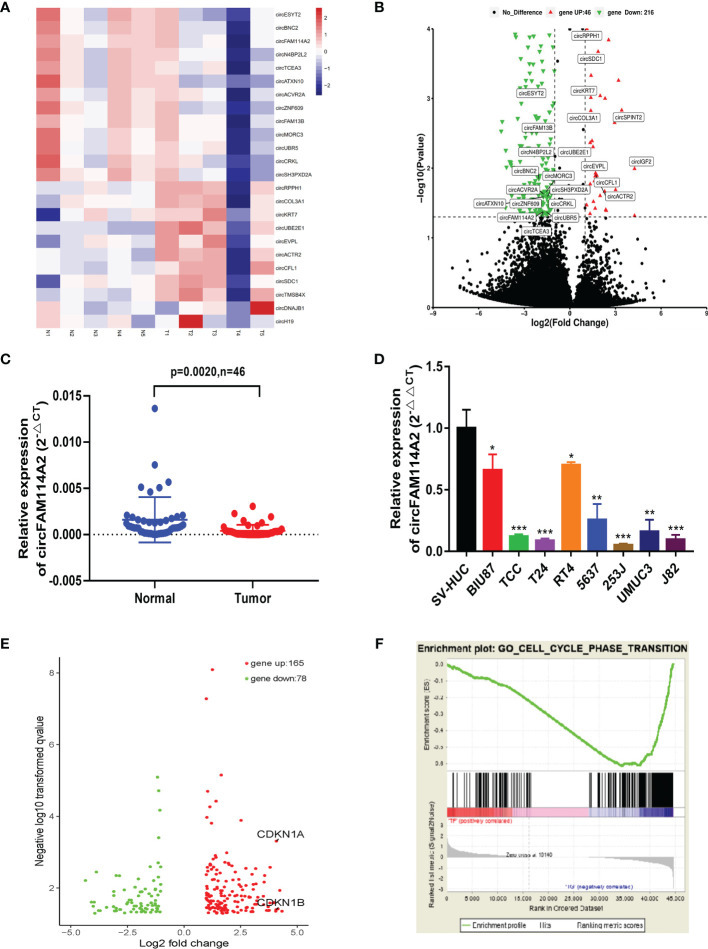
CircFAM114A2 was downregulated in urothelial carcinoma and associated with cell cycle regulation pathways. **(A)** Heat map of RNA sequencing in five pairs of urothelial carcinoma tissues. **(B)** CircRNAs with different expression levels were screened by fold change and *p*-value. **(C)** Quantitative real-time PCR (qRT-PCR) assay with divergent primers confirmed the low expression of circFAM114A2 in 46 pairs of human urothelial carcinoma tissues compared with their adjacent normal tissues. **(D)** The expressions of circFAM114A2 in SV-HUC and urothelial carcinoma cell lines were measured by qRT-PCR (Student’s *t*-test: *p < 0.05, **p < 0.01, ***p < 0.001). **(E)** Genes with different expression levels screened by fold change and *p*-value in mRNA sequencing. **(F)** Gene set enrichment analysis (GSEA) showed that the enrichment occurred in the cell cycle transition-related genes, including *P27* and *P21*.

RNA sequencing on five pairs of urothelial carcinoma tissues was carried out and differentially expressed circRNAs were screened out (**Figure 1A**). The downregulated circRNAs were sorted by fold change of more than 2 and a *p*-value of <0.05 and then were screened with an average read count of normal tissue more than 150. We found that circFAM114A2 was one of the three most downregulated circRNAs (**Figure 1B**). Among them, circFAM114A2 had a much higher tissue average read count than the other two circRNAs. The qRT-PCR results showed that circFAM114A2 was significantly reduced in 46 paired urothelial carcinoma tissues (**Figure 1C**) and eight urothelial carcinoma lines (BIU87, TCC, RT4, 5637, T24, J82, UMUC, and 253J) in comparison to SV-HUC, a healthy urothelial cell line (**Figure 1D**). Additionally, we also found that FAM114A2 mRNA was downregulated in urothelial carcinoma tissue (**Supplementary Figure S1A**) and cell lines (**Supplementary Figure S1B**). We validated head-to-tail splicing through qRT-PCR of circFAM114A2 using an estimated size established by Sanger sequencing (**Supplementary Figure S2A**). Then, the convergent primers to increase FAM114A2 mRNA and divergent primers to amplify circFAM114A2 were designed. CircFAM114A2 could only be amplified by divergent primers in cDNA rather than gDNA (**Supplementary Figure S2B**). We additionally showed that FAM114A2 mRNA was drastically decreased post-RNase R addition, although circFAM114A2 was impervious to RNase R (**Supplementary Figure S2C**). Patients with different circFAM114A2 expression values (mean value = 0.000125103) were divided into low- and high-circFAM114A2 groups. We found that the levels of circFAM114A2 were negatively associated with histological grade (*p* = 0.02) (**Table 1**). We predicted the recurrence and progression in NMIBC patients and stratified the risk group with the European Organisation for Research and Treatment of Cancer (EORTC) method (22). We found that the recurrence scores were lower in the high-circFAM114A2 expression group (**Table 1**). In addition, urothelial carcinoma patients with higher circFAM114A2 levels had better overall survival (**Supplementary Figure S2D**). To examine the role of circFAM114A2 in urothelial carcinoma, we separately chose a high malignant urothelial carcinoma cell line, T24, and low malignant urothelial carcinoma cell line, 5637, for further functional assays. We transfected the circFAM114A2 overexpression or knockdown lentivirus into T24 and 5637 cells. We discovered that the levels of circFAM114A2 were substantially changed, while the levels of FAM114A2 mRNA had no significant change (**Supplementary Figures S2E, F**). In three pairs of circFAM114A2 overexpression and negative control cells, mRNA sequencing and enrichment analysis (gene seat enrichment analysis, GSEA) were performed (**Figure 1E**). We found that the cell cycle transition genes were significantly related to the expression of circFAM114A2 (**Figure 1F**).

**Table 1:** Correlations between the expression of circFAM114A2 and clinicopathological features in urothelial carcinoma patients.

Characteristics	Case	circ-FAM114A2		*p*-value
		Low	High	
All cases	46	28	18	
Age (years)				0.152
<65	20	12	4	
≥65	26	16	14	
Gender				0.834
Male	34	21	13	
Female	12	7	5	
TNM stage				0.113
Ta–T1	12	5	7	
T2–T4	34	23	11	
Urothelial carcinoma type				0.113
NMIBC	12	5	7	
MIBC	34	23	11	
Histological grade				0.020*
Low	10	3	7	
High	36	25	11	
Histological grade				0.043*
G1	2	0	2	
G2	10	4	6	
G3	34	24	10	
Tumor size (cm)				0.080
<3	16	7	9	
≥3	30	21	9	
Recurrence score				0.023*
0	0	0	0	
1–4	0	0	0	
5–9	7	1	6	
10–17	5	4	1	
Progression score				0.424
0	0	0	0	
2–6	2	0	2	
7–13	4	2	2	
14–23	6	3	3	

TNM, tumor node metastasis; NMIBC, non-muscle invasive bladder cancer; MIBC, muscle invasive bladder cancer.

*p < 0.05.

### CircFAM114A2 Induced Cell Cycle Arrest and Enhanced the Cisplatin Chemosensitivity of Urothelial Carcinoma Cells *In Vitro*

**Figure 2:**
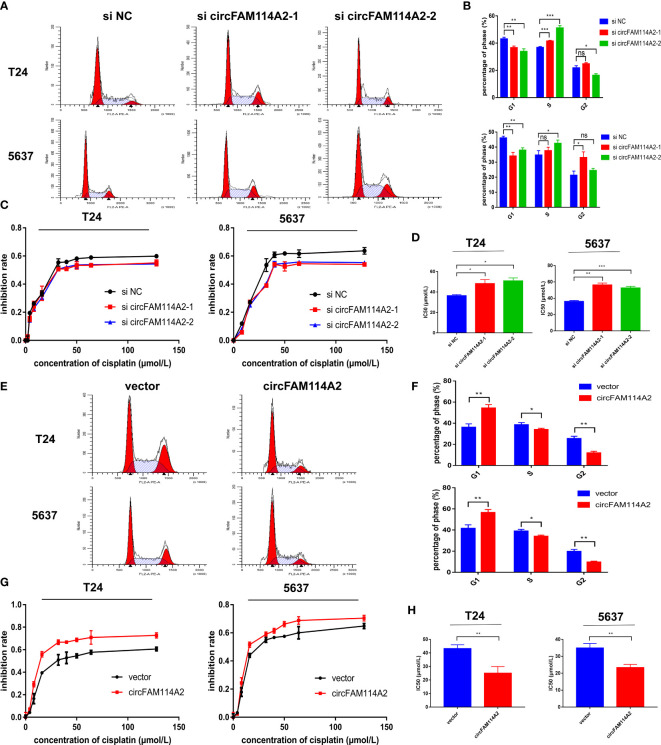
CircFAM114A2 induced cell cycle arrest and enhanced the cisplatin chemosensitivity of urothelial carcinoma cells *in vitro*. **(A)** Illustrations of the flow cytometry assays in circFAM114A2 knockdown T24 and 5637 cells. **(B)** Fewer cells were distributed in the G1 phase and more cells were distributed in the S phase of the circFAM114A2 knockdown group compared with the control group in T24 and 5637 cell lines (Student’s *t*-test: *p < 0.05, **p < 0.01, ***p < 0.001). ns, no significant. **(C)** The cell viability curve of the cisplatin treatment assays in the circFAM114A2 knockdown group and relative control group. **(D)** IC_50_ values showed that circFAM114A2 knockdown could reduce the sensitivity of T24 and 5637 cells to cisplatin (Student’s *t*-test: *p < 0.05, **p < 0.01, ***p < 0.001). **(E)** Illustrations of the flow cytometry assays in circFAM114A2 overexpression T24 and 5637 cells. **(F)** More cells were distributed in the G1 phase and fewer cells were distributed in the S phase in the circFAM114A2 overexpression group compared with the control group in T24 and 5637 cells (Student’s *t*-test: *p < 0.05, **p < 0.01, ***p < 0.001). **(G)** The cell viability curve of the cisplatin treatment assays in the circFAM114A2 overexpression group and relative control group. **(H)** IC_50_ values showed that the overexpression of circFAM114A2 could increase the sensitivity of T24 and 5637 cells to cisplatin (Student’s *t*-test: **p < 0.01, ***p < 0.001). Data are the mean ± SD, *n* = 3.

Flow cytometry results indicated that fewer cells were dispersed in the G1 phase of the circFAM114A2 knockdown group (**Figures 2A, B** **)**. In order to reveal the role of circFAM114A2 in cisplatin chemosensitivity, we designed 10 gradient cisplatin diluted concentrations (0, 2, 4, 8, 16, 32, 40, 50, 64, and 128 µmol/L). The cell viability inhibition curves and the IC_50_ values indicated that, in the circFAM114A2 knockdown group, the cisplatin sensitivity decreased (**Figures 2C, D** **)**. More cells were dispersed in the G1 phase of the circFAM114A2 overexpression group compared with the vector group (**Figures 2E, F** **)**, which implied that circFAM114A2 stimulated G1 cell cycle arrest. The cell viability inhibition curves and the IC_50_ values indicated that circFAM114A2 overexpression may drastically constrain the growth of T24 and 5637 cells across different diluted concentrations of cisplatin compared to the vector group (**Figures 2G, H** **)**. Through the CCK-8 assay, the cell cloning formation experiment, and xenografts in nude mice, we found that circFAM114A2 could also inhibit the proliferation of urothelial carcinoma cells *in vitro* and *in vivo* (**Supplementary Figures S3, S4**). Additionally, apoptosis experiments indicated that circFAM114A2 induced cell apoptosis in urothelial carcinoma cells (**Supplementary Figure S5**). The overexpression of circFAM114A2 induced a higher apoptosis rate compared to the vector group with cisplatin treatment (**Supplementary Figure S5**).

### CircFAM114A2 Acted as a Molecular Sponge for miR-222-3p and miR-146a-5p

**Figure 3:**
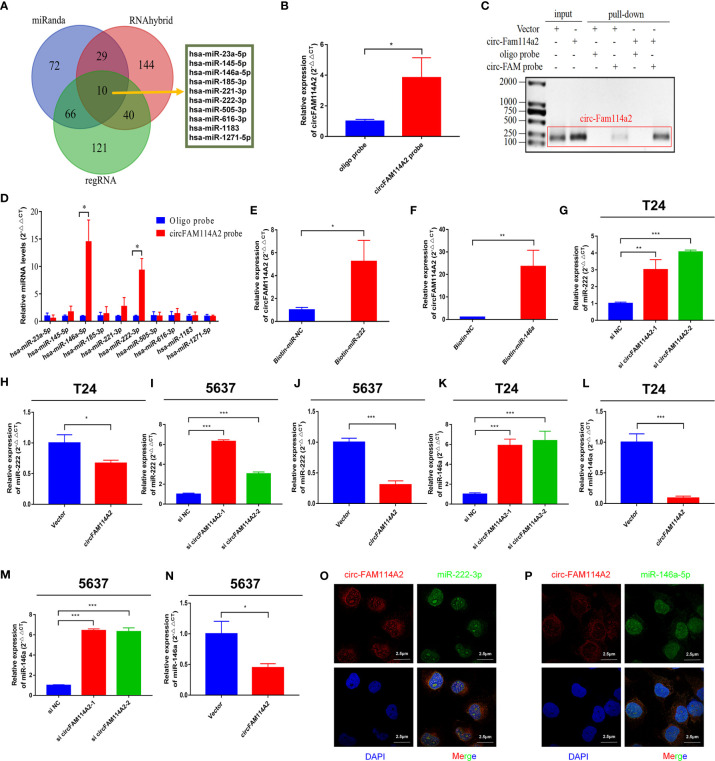
CircFAM114A2 acted as a sponge for miR-222-3p and miR-146a-5p in urothelial carcinoma cells. **(A)** Schematic illustration showed overlapping of the target miRNAs of circFAM114A2 predicted by miRanda, regRNA, and RNAhybrid. **(B)** CircFAM114A2 was pulled down from T24 lysates after transfection with the circFAM114A2 biotinylated probe and investigated with quantitative real-time PCR (qRT-PCR) (Student’s *t*-test: *p < 0.05). **(C)** CircFAM114A2 in urothelial carcinoma cell lysates was pulled down and enriched with a circFAM114A2-specific probe and then detected by qRT-PCR. The amplification products were confirmed by agarose gel electrophoresis. The relative level of circFAM114A2 was normalized to input. **(D)** The relative levels of 10 miRNA candidates in the urothelial carcinoma cell lysates were detected by qRT-PCR (Student’s *t*-test: *p < 0.05). **(E, F)** Biotin-coupled miR-222-3p/miR-146a-5p captured a fold change of circFAM114A2 in the complex as compared with biotin-coupled nonsense control (NC) in biotin-coupled miRNA capture (Student’s *t*-test: *p < 0.05, **p < 0.01). **(G, H)** The expression level of miR-222-3p was detected by qRT-PCR in circFAM114A2 knockdown and overexpression T24 cells (Student’s *t*-test: *p < 0.05, **p < 0.01, ***p < 0.001). **(I, J)** The expression level of miR-222-3p was detected by qRT-PCR in circFAM114A2 knockdown and overexpression 5637 cells (Student’s *t*-test: ***p < 0.001). **(K, L)** The expression level of miR-146a-5p in circFAM114A2 knockdown and overexpression T24 cells was detected by qRT-PCR (Student’s *t*-test: ***p < 0.001). **(M, N)** The expression level of miR-146a-5p was detected by qRT-PCR in circFAM114A2 knockdown and overexpression 5637 cells (Student’s *t*-test: *p < 0.05, ***p < 0.001). **(N)** Expression level of miR-146a-5p in circFAM114A2 overexpression 5637 cells. **(O, P)** RNA fluorescence *in situ* hybridization (FISH) for circFAM114A2 and miR-222-3p/miR-146a-5p was detected in T24. Nuclei was stained *blue* (DAPI), circFAM114A2 was stained *red*, and miR-222-3p/miR-146a-5p were stained *green*. Data are the mean ± SD, *n* = 3.

In order to investigate whether circFAM114A2 may function as a sponge for miRNAs in urothelial carcinoma cells, three publicly accessible predictive algorithms—miRanda (http://www.miranda.org/) and (http://regrna2.mbc.nctu.edu.tw/), and RNAhybrid (https://bibiserv.cebitec.uni-bielefeld.de/rnahybrid/)—were used to forecast the potential binding miRNAs of circFAM114A2. Overall, 10 potential miRNAs were predicted by the three prediction instruments (**Figure 3A**). Subsequently, we detected the expressions of 10 potential miRNAs with the biotin-labeled probe pull-down tests. The biotin-labeled probe was confirmed to bring down circFAM114A2 in T24 cells (**Figures 3B, C** **)**. The results indicated that only miR-222-3p and miR-146a-5p were abundantly pulled down by circFAM114A2 (**Figure 3D**). To additionally verify the sponge effect of circFAM114A2, we administered biotin-labeled miR-222-3p and miR-146a-5p mimics to confirm the direct binding of these miRNAs to circFAM114A2. As the data indicated, the biotin-labeled miR-222-3p/miR-146a-5p mimics detained additional circFAM114A2 compared to the biotin-labeled negative control (**Figures 3E, F** **)**. We examined the level of miR-222-3p/miR-146a-5p in T24 and 5637 urothelial carcinoma cells, which had different expression levels of circFAM114A2. These results showed that the expressions of miR-222-3p and miR-146a-5p in T24 and 5637 cells were increased by circFAM114A2 knockdown, while the overexpression of circFAM114A2 reduced the miR-222-3p and miR-146a-5p expressions in T24 and 5637 cells (**Figures 3G–N**). Furthermore, RNA fluorescence *in situ* hybridization (FISH) experiments indicated that circFAM114A2 and miR-222-3p/miR-146a-5p co-localized in the cytoplasm and the nucleus (**Figures 3O, P** **)**. Overall, these results proved that circFAM114A2 could bind to both miR-222-3p and miR-146a-5p.

### MIR-222-3p/146a-5p Attenuated Cell Cycle Arrest and Decreased Cisplatin Chemotherapy Sensitivity

**Figure 4:**
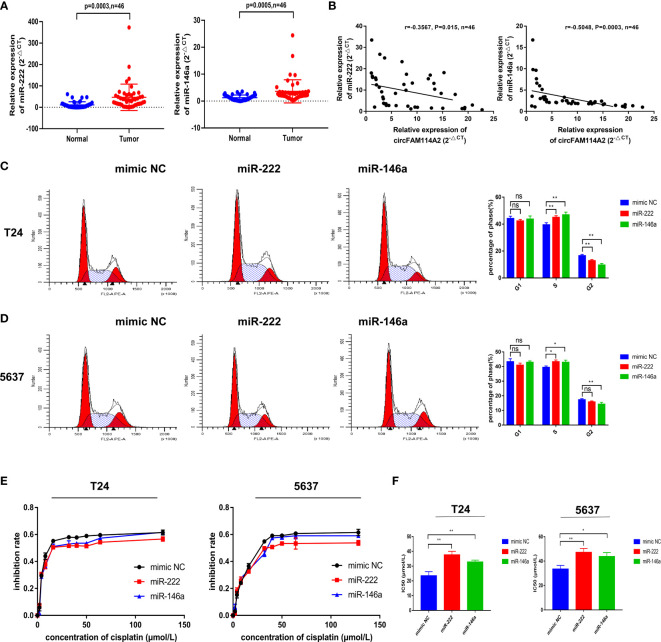
MIR-222-3p/146a-5p attenuated cell cycle arrest and decreased cisplatin chemotherapy sensitivity. **(A)** Quantitative real-time PCR (qRT-PCR) revealed that miR-222-3p and miR-146a-5p were upregulated in urothelial carcinoma tissues (*n* = 46) compared with normal adjacent bladder tissues. **(B)** A negative correlation between the expressions of circFAM114A2 and miR-222-3p/miR-146a-5p was shown using Pearson’s correlation analysis. **(C, D)** Illustrations of the flow cytometry assays in miR-222-3p/miR-146a-5p mimic transfected T24 and 5637 cells. More cells were distributed in the S phase of the miR-222-3p/miR-146a-5p mimic group compared with the control group in T24 and 5637 cell lines (Student’s *t*-test: *p < 0.05, **p < 0.01). ns, no significant. **(E)** Cell viability curve of the cisplatin treatment assays in the miR-222-3p/miR-146a-5p mimic group and relative control group. **(F)** IC_50_ values showed that the overexpression of miR-222-3p and miR-146a-5p could decrease the sensitivity of T24 and 5637 cells to cisplatin (Student’s *t*-test: *p < 0.05, **p < 0.01). Data are the mean ± SD, *n* = 3.

We found that miR-222-3p and miR-146a-5p had high expressions in 46 urothelial carcinoma tissues in comparison to adjacent normal tissues (**Figure 4A**). A negative relationship among the levels of circFAM114A2 and miR-222-3p/miR-146a-5p was indicated utilizing Pearson’s correlation analysis (**Figure 4B**). The transfection of the miR-222-3p/miR-146a-5p mimics in T24 and 5637 cells could arrest the cell cycle in the S phase by flow cytometry (**Figures 4C, D** **)**. In order to confirm the roles of miR-222-3p and miR-146a-5p in cisplatin chemosensitivity, we designed 10 gradient cisplatin diluted concentrations (0, 2, 4, 8, 16, 32, 40, 50, 64, and 128 µmol/L). The cell viability inhibition curves and the IC_50_ values indicated that miR-222-3p and miR-146a-5p could inhibit the cisplatin sensitivity of T24 and 5637 cells (**Figures 4E, F** **)**.

### MIR-222-3p/146a-5p Interference Rescued the Cell Cycle Arrest and Cisplatin Chemosensitization Induced by circFAM114A2 in Urothelial Carcinoma Cells

**Figure 5:**
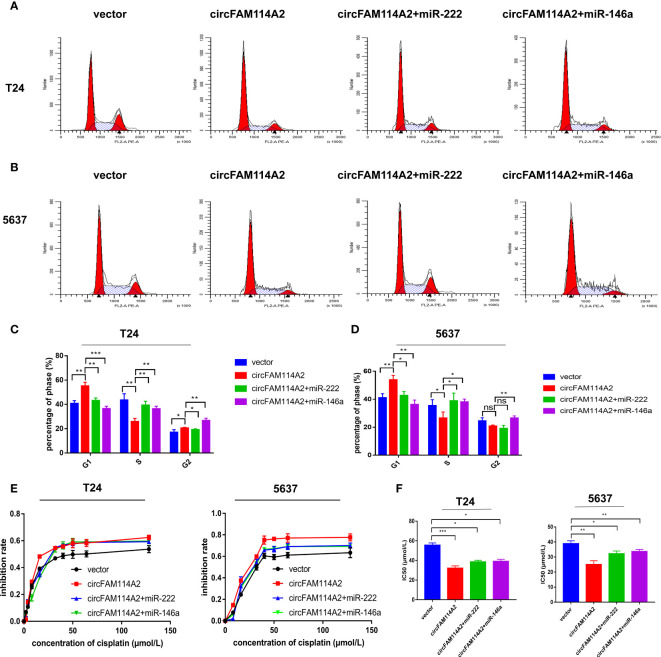
MIR-222-3p/146a-5p interference rescued the cell cycle arrest and cisplatin chemosensitization induced by circFAM114A2 in urothelial carcinoma cells. **(A)** Illustrations of the flow cytometry assays in T24 cells. **(B)** Illustrations of the flow cytometry assays in 5637 cells. **(C)** More cells were distributed in the G1 phase and fewer cells were distributed in the S phase in the circFAM114A2 overexpression group compared with the control group in T24 cells, and miR-222-3p and miR-146a-5p mimic transfection could reverse the G1 phase redundant and reduced S phase (Student’s *t*-test: *p < 0.05, **p < 0.01, ***p < 0.001). **(D)** More cells were distributed in the G1 phase and fewer cells were distributed in the S phase in the circFAM114A2 overexpression group compared with the control group in 5637 cells, and miR-222-3p and miR-146a-5p mimic transfection could reverse the G1 phase redundant and reduced S phase (Student’s *t*-test: *p < 0.05, **p < 0.01). ns, no significant. **(E)** The cell viability curve of the cisplatin treatment assays showed that miR-222-3p/miR-146a-5p mimics could reverse the high inhibition rate induced by circFAM114A2 in T24 and 5637 cells. **(F)** IC_50_ values showed that co-transfection with the miR-222-3p or miR-146a-5p mimic could reverse the increased sensitivity to cisplatin induced by circFAM114A2 in T24 and 5637 cells (Student’s *t*-test: *p < 0.05, **p < 0.01, ***p < 0.001). Data are the mean ± SD, *n* = 3.

The rescue experiment was performed by co-transfecting circFAM114A2 and the miR-222-3p/miR-146a-5p mimics into urothelial carcinoma cells. Transfection of the miR-222-3p/miR-146a-5p mimics in circFAM114A2 overexpression cells could rescue the G1 cell cycle arrest phenomenon induced by circFAM114A2 (**Figures 5A–D**). The results from the CCK-8 assay suggested that the progression of urothelial carcinoma cell growth was appreciably hindered when treated with different diluted concentrations (0, 2, 4, 8, 16, 32, 40, 50, 64, and 128 µmol/L) of cisplatin by the overexpression of circFAM114A2, while the ectopic expression of miR-222-3p or miR-146a-5p could partly attenuate this phenomenon (**Figures 5E, F** **)**. More than that, the overexpression of miR-222-3p or miR-146a-5p substantially encouraged the proliferation of T24 and 5637 cells (**Supplementary Figures S6A, B**). CCK-8 and the colony formation assay indicated that a high expression of circFAM114A2 was associated with an inhibition in growth. However, this influence may, in part, be inhibited by ectopic miR-222-3p or miR-146a-5p expression (**Supplementary Figures S6C–F**). All these data confirmed that miR-222-3p/146a-5p could rescue the inhibition of progression and cisplatin chemosensitization induced by circFAM114A2 in urothelial carcinoma cells.

### CircFAM114A2 Influenced the Expressions of *P27* and *P21* Through Sponging miR-222-3p and miR-146a-5p

**Figure 6:**
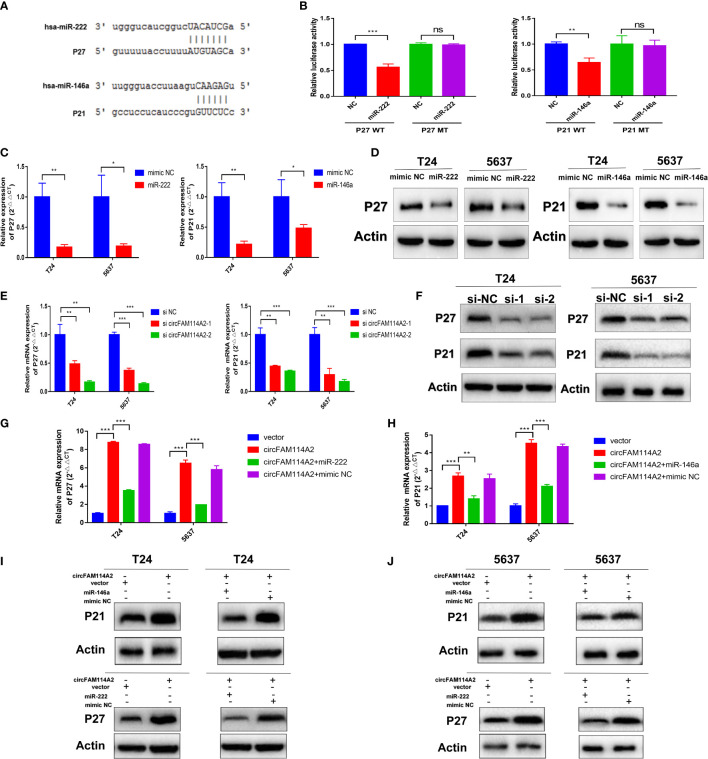
CircFAM114A2 influenced the expressions of *P27* and *P21* through sponging miR-222-3p and miR-146a-5p. **(A)** Predicted miR-222-3p/miR-146a-5p binding sites in the 3′-UTRs of *P27*/*P21* mRNA by bioinformatics analysis. **(B)** Dual-luciferase reporter assays demonstrated that *P27* and *P21* are direct targets of miR-222-3p and miR-146a-5p, respectively (Student’s *t*-test: **p < 0.01, ***p < 0.001). ns, no significant. **(C)** MiR-222-3p/miR-146a-5p could reduce the expressions of *P27*/*P21* in T24 and 5637 cells detected by quantitative real-time PCR (qRT-PCR) (Student’s *t*-test: *p < 0.05, **p < 0.01). **(D)** MiR-222-3p/miR-146a-5p could reduce the expressions of *P27*/*P21* in T24 and 5637 cells detected by Western blot. **(E)** qRT-PCR results indicated that the knockdown of circFAM114A2 downregulated the mRNA expression levels of *P27* and *P21* in T24 and 5637 cells (Student’s *t*-test: **p < 0.01, ***p < 0.001). **(F)** Western blot results showed that the knockdown of circFAM114A2 downregulated the protein expression levels of *P27* and *P21*. **(G, H)** Overexpression of circFAM114A2 upregulated the mRNA expression levels of *P27* and *P21* in T24 and 5637 cells, which could be reversed by co-transfection with miR-222-3p and miR-146a-5p, respectively (Student’s *t*-test: **p < 0.01, ***p < 0.001). **(I, J)** Overexpression of circFAM114A2 upregulated the protein expression levels of *P27* and *P21*, which could be reversed by co-transfection with miR-222-3p and miR-146a-5p, respectively. Data are the mean ± SD, *n* = 3.

In accordance with miRanda and TargetScan, we predicted that miR-222-3p and miR-146a-5p targeted the 3′-UTRs of *P27* and *P21*, respectively (**Figure 6A**). Dual-luciferase reporter assay was carried out to confirm miR-222-3p/miR-146a-5p binding with *P27*/*P21*. The wild-type and mutant 3′-UTRs of *P27* and *P21* were added to the construct reporter plasmids. Co-transfection of the miR-222-3p mimic and the wild-type P27 reporter plasmid reduced the luciferase activity dramatically. Co-transfection of miR-146a-5p and the wild-type P21 reporter plasmid also reduced the luciferase activity (**Figure 6B**). On the contrary, co-transfection with the miR-222-3p or miR-146a-5p mimic and the mutated P27 or P21 vector indicated no significant influence on the luciferase activity (**Figure 6B**). The qRT-PCR and Western blot results revealed that miR-222-3p reduced the expression of *P27* and that miR-146a-5p reduced the expression of *P21* (**Figures 6C, D** and **Supplementary Figure S7**). These results implied that these two pathways are two separate pathways. Knockdown of circFAM114A2 could decrease *P27* and *P21* mRNA and protein (**Figures 6E, F** **)**. Consequently, high levels of circFAM114A2 may heighten the mRNA and protein expressions of *P27* and *P21* (**Figures 6G–J**). On the other hand, co-transfection with the miR-222-3p or miR-146a-5p mimic and the circFAM114A2 overexpression lentivirus partly inhibited the increased expressions of *P27* and *P21* induced by circFAM114A2 (**Figures 6G–J**). All these findings confirmed that *P27* and *P21* were directly regulated by miR-222-3p and miR-146a-5p, respectively.

### CircFAM114A2 Promoted Cisplatin Sensitivity *In Vivo*

**Figure 7:**
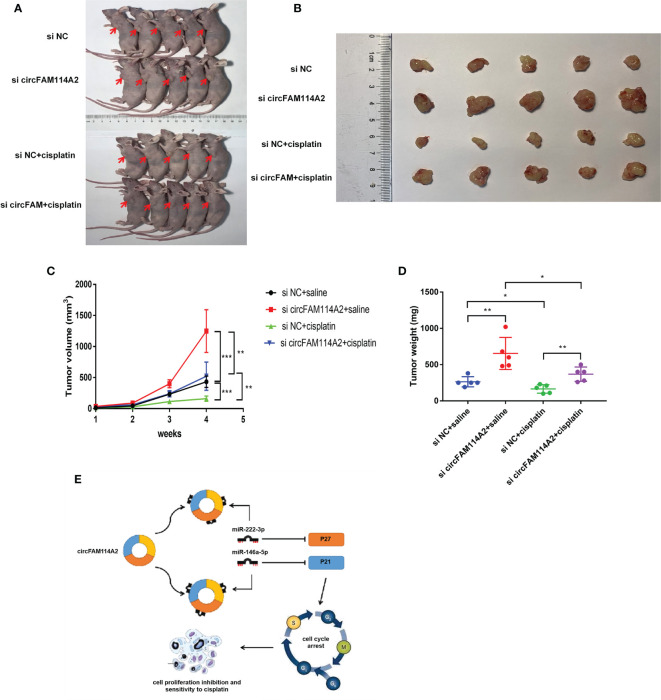
CircFAM114A2 promoted cisplatin sensitivity in nude mice. **(A)** Representative image of nude mice injected with si circFAM114A2 or si NC transfection T24 cells, which were treated with cisplatin or saline (*n* = 5). **(B)** Representative picture of the tumor formation of xenografts in nude mice injected with si circFAM114A2 or si NC transfection T24 cells, which were treated with cisplatin or saline (*n* = 5). **(C)** The tumor volumes of nude mice were measured every week (Student’s *t*-test: **p < 0.01, ***p < 0.001). **(D)** The weights of the tumors in the four groups were measured using electronic scales (Student’s *t*-test: *p < 0.05, **p < 0.01). **(E)** Mode pattern of the circFAM114A2-miR-222-3p/miR-146a-5p-P27/P21 regulatory network.

To investigate whether circFAM114A2 could influence the sensitivity of cisplatin chemotherapy *in vivo*, T24 cells transfected with si circFAM114A2 or si NC were inoculated in nude mice subcutaneously. One week after inoculation, nude mice in the experimental groups were intraperitoneally injected with cisplatin (5 mg/kg) every 3 days, while the control groups were injected with the same volume of saline. After 4 weeks, we observed the tumor in nude mice and removed it (**Figures 7A, B** **)**. The tumor weight and volume of the si circFAM114A2 group treated with cisplatin were higher compared to those of the si NC group treated with cisplatin (**Figures 7C, D** **)**. All the results indicated that circFAM114A2 could promote cisplatin sensitivity *in vivo*.

## Discussion

In recent years, circRNAs have been increasingly shown to act as vital factors in the incidence and treatment of urothelial carcinoma (6, 23, 24). We detected circFAM114A2 through circRNA sequencing technology and confirmed that circFAM114A2 was decreased in urothelial carcinoma tissues and cell lines, consistent with a study by Liu et al. (25) Studies of circFAM114A2 in other tumors are still lacking. The expressions of many circRNAs, including circFAM114A2, were reduced in tumor cells. CircRNA formation originates from specific splicing of pre-mRNA. Wei et al. reported that the downregulation of EIF4A3 could suppress the transcription of circRNA in cancer cells (26). Zhang et al. reported that methylation of the flanking introns of pre-mRNA could promote the formation of relative circRNA (27). Therefore, the expression level of circRNA was also influenced by the methylation process. All in all, the downregulation of circFAM114A2 in cancer cells may be induced by methylation or, simply, transcriptional suppression. Furthermore, we found that circFAM114A2 blocked more urothelial carcinoma cells into the G1 phase and inhibited cisplatin chemoresistance by eliminating the effects of miR-222-3p and miR-146a-5p. In this study, we revealed two axes of circFAM114A2-miR-222-3p-P27 and circFAM114A2-miR-146a-5p-P21 that played an important role in the inhibition of urothelial carcinoma progression and cisplatin chemoresistance. A working model is shown in **Figure 7E**.

Cisplatin always has a vital function in the chemotherapy of urothelial carcinoma. The long-term survival of patients is greatly hindered due to cisplatin resistance (28). Previously, Yuan et al. reported that circCDR1as could improve the sensitivity of urothelial carcinoma to cisplatin through increasing the APAF1 levels by inhibiting miR-1270 (6). Our research revealed the cell cycle arrest and chemosensitization functions of circFAM114A2 in urothelial carcinoma. The flow cytometry results indicated that circFAM114A2 inhibited cell cycle transition and blocked more urothelial carcinoma cells in the G1 phase. The CCK-8 assay indicated that circFAM114A2 could drastically block the growth of urothelial carcinoma cells across different diluted concentrations of cisplatin. As reported, cell cycle regulation and cell apoptosis are the theoretical basis of cisplatin action and play important roles in cisplatin chemotherapy resistance (14, 15). It has been reported that tumors are most sensitive to cisplatin in the G1 phase (29, 30). Therefore, blocking tumor cells in the G1 phase can enhance their sensitivity to cisplatin. In our study, we found that a high expression of circFAM114A2 could block more urothelial carcinoma cells in the G1 phase. Furthermore, we discovered that a high expression of circFAM114A2 may heighten the apoptosis rate of urothelial carcinoma cells. Studies have shown that circRNAs can influence the apoptosis levels of gastric cancer, urothelial carcinoma, and non-small cell lung cancer cells (31–33). Hence, our results suggested that improved sensitivity induced by circFAM114A2 may also relate to a high apoptosis rate.

CircRNAs can not only regulate the sensitivity of cisplatin chemotherapy but also affect the sensitivity of many other chemotherapy drugs. CircCRIM1 could promote docetaxel chemoresistance in nasopharyngeal carcinoma (34). CircNFIX could inhibit temozolomide chemotherapy sensitivity in glioma (35). CircRNAs also played an important regulatory role in immunotherapy (36). CircRNAs can regulate the immune function and affect the occurrence and development of tumors, which provide more enlightenment for the immunotherapy of different tumors (37). It had been reported that CircMET can lead to immunosuppression and resistance to anti-PD1 therapy in hepatocellular carcinoma (38). In addition, circRNA can be enriched in exosomes and further affect the sensitivity to immunotherapy (37). Zhang et al. reported that tumor cell-derived exosomal circRNA UHRF1 could enhance anti-PD1 therapy resistance in hepatocellular carcinoma (37). With more further studies on the correlation between circRNA and chemosensitivity appearing, discovery of new targets for the treatment of tumors in the future is expected.

In summary, circFAM114A2 potentially has a function in predicting cisplatin chemosensitivity in urothelial carcinoma patients.

## Data Availability Statement

The original contributions presented in the study are included in the article/**Supplementary Material**. Further inquiries can be directed to the corresponding authors.

## Ethics Statement

The studies involving human participants were reviewed and approved by The First Affiliated Hospital of Nanjing Medical University. The patients/participants provided written informed consent to participate in this study. The animal study was reviewed and approved by The First Affiliated Hospital of Nanjing Medical University.

## Author Contributions

QL and HY conceived and designed the study. JL, ZZ, JW, HY, JH, DF, BY, QW, and PL performed the experiments. JL and XY wrote the paper. QL and HWY reviewed and edited the manuscript. All authors contributed to the article and approved the submitted version.

## Funding

This work was supported by the National Natural Science Foundation of China (nos. 81772711; 82073306; 82072832; 81602235), Jiangsu Province’s Key Provincial Talents Program (ZDRCA2016006), the “333” Project of Jiangsu Province (LGY2016002 and 2018055), the Provincial Initiative Program for Excellency Disciplines of Jiangsu Province (no. BE2016791), Postgraduate Research & Practice Innovation Program of Jiangsu Province (KYCX20_1397; KYCX21_1607), and the Priority Academic Program Development of Jiangsu Higher Education Institutions (PAPD).

## Conflict of Interest

The authors declare that the research was conducted in the absence of any commercial or financial relationships that could be construed as a potential conflict of interest.

## Publisher’s Note

All claims expressed in this article are solely those of the authors and do not necessarily represent those of their affiliated organizations, or those of the publisher, the editors and the reviewers. Any product that may be evaluated in this article, or claim that may be made by its manufacturer, is not guaranteed or endorsed by the publisher.

## Abbreviations

circRNAs, circular RNAs; MIBC, muscle invasive bladder cancer; NMIBC, non-muscle invasive bladder cancer; ncRNA, non-coding RNA; ceRNA, competitive endogenous RNA; NC, nonsense control; PBS, phosphate-buffered saline; FISH, fluorescence *in situ* hybridization; TNM, tumor node metastasis.
