# Supplementary material for: CircFAM114A2 Promotes Cisplatin Sensitivity via miR-222-3p/P27 and miR-146a-5p/P21 Cascades in Urothelial Carcinoma
Source: Front Oncol. 2021 Oct 14;11:659166. doi: 10.3389/fonc.2021.659166 (PMC8551855; doi:10.3389/fonc.2021.659166)
Supplement: Supplementary file 1 [file DataSheet_1.docx]

**Supporting Information：**

**Supplementary Figures**

**Figure S1: FAM114A2 was down-regulated in urothelium carcinoma tissues and cell lines**

1. B QRT-PCR assay showed that FAM114A2 mRNA was down-regulated in 46 pairs of human urothelium carcinoma tissues compared with their adjacent normal tissues and cell lines

**Figure S2: CircFAM114A2 was correlated with prognosis of patients and validation of circFAM114A2 overexpression and knockdown efficiency**

**A** Schematic illustration showed the circularization of FAM114A2 exon 2, exon 3 and exon 4 forming circFAM114A2. The existence of circFAM114A2 was proved by RT-PCR and back splicing junction was verified by Sanger sequencing. Gray arrow indicates the special splicing junction of circFAM114A2. **B** The existence of circFAM114A2 was validated in sv-huc, 5637 and T24 cell lines by RT-PCR. Divergent primers amplified circFAM114A2 in cDNA but not genomic DNA (gDNA). β-actin was used as negative control. **C** The expression of circFAM114A2 and FAM114A2 mRNA in T24 and 5637 cells treated with or without RNase R were detected by qRT-PCR (***P<0.001, Student’s t-test). **D** Kaplan-Meier plotter analysis of the correlation of circFAM114A2 expression level with overall survival of urothelium carcinoma patients. **E** Two siRNAs vectors specifically targeting circFAM114A2 and control vector (NC) were transfected into T24 and 5637 cells respectively. The interfering efficacy of each siRNA vector on circFAM114A2 and FAM114A2 mRNA was tested by qRT-PCR (***P<0.001, Student’s t-test). **F** The expression levels of circFAM114A2 and FAM114A2 mRNA in T24 and 5637 cells transfected with circFAM114A2 or control vector plasmids were detected by qRT-PCR (****P*<0.001, Student’s t-test). Data are mean±SD, n=3

**Figure S3:** **CircFAM114A2 could inhibit urothelium carcinoma proliferation *in vitro***

**A, B** Knockdown of circFAM114A2 promoted cell proliferation as indicated by CCK-8 assays in T24 and 5637 cells (**P*<0.05, ***P*<0.01, Student’s t-test). **C, D** Colony formation assay showed that circFAM114A2 knockdown significantly increased the cloning number of T24 and 5637 cells compared with control group (***P*<0.01, ****P*<0.001, Student’s t-test). **E, F** Overexpression of circFAM114A2 inhibited cell proliferation as indicated by CCK-8 assays in T24 and 5637 cells (***P*<0.01, ****P*<0.001, Student’s t-test). **G, H** Colony formation assay showed that overexpression of circFAM114A2 significantly decreased the cloning number of T24 and 5637 cells compared with control group (***P*<0.01, ****P*<0.001, Student’s t-test). Data are mean±SD, n=3

**Figure S4: CircFAM114A2 suppressed tumor formation of xenograft in nude mice**

**A** Representative image of the nude mice injected with circFAM114A2 or vector transfection T24 cells (n=4). **B** Representation picture of tumor formation of xenograft in nude mice injected with circFAM114A2 or vector transfection T24 cells (n=4). **C** Weights of tumors in two groups were measured using electronic scales (***P*<0.01, Student’s t-test). **D** Summery of tumor volume of mice which were measured every week (****P*<0.001, Student’s t-test). **E** The expression of P27/P21 was also measured using IHC in xenograft (***P*<0.01, Student’s t-test, Magnification,×40). **F** and **G** The verification of miR-222-3p and miR-146a-5p in 4 pairs of xenograft tumors transfected with circFAM114A2 or vector detected by qRT-PCR (**P*<0.05, ***P*<0.01, Student’s t-test). **H**. The level of P27/P21 expression in 4 pairs of xenograft tumors transfected with circFAM114A2 or vector was detected by western blot.

**Figure S5: Overexpression of circFAM114A2 increased the apoptotic rate of urothelium carcinoma cells**

1. **D** Overexpression of circFAM114A2 could increase the apoptotic rate of T24 and 5637 cells. urothelium carcinoma cells with high expression of circFAM114A2 treated with cisplatin had more apoptotic cells compared with vector group (**P*<0.05, ***P*<0.01, Student’s t-test). Data are mean±SD, n=3

**Figure S6: MIR-222-3p/miR-146a-5p played as oncogenes and transfection of miR-222-3p/miR-146a-5p mimic eliminated the repression function of circFAM114A2**

**A, B** CCK-8 assay showed that miR-222-3p and miR-146a-5p promoted the proliferation of T24 and 5637 cells (**P*<0.05, ****P*<0.001, Student’s t-test). **C, D** CCK-8 assays showed that co-transfected with miR-222-3p or miR-146a-5p mimic could reverse proliferation-repression function of circFAM114A2 in T24 and 5637 cells (***P*<0.01, ****P*<0.001, Student’s t-test). **E, F** Colony formation assay showed that co-transfecting with miR-222-3p or miR-146a-5p mimic could reverse the decreasing cloning number of T24 and 5637 cells caused by circFAM114A2 (***P*<0.01, ****P*<0.001, Student’s t-test). Data are mean±SD, n=3

**Figure S7: The exploration of the relationship between the two pathways**

**A, B** The transfection of miR-222-3p mimic could not influence the expression of P21 detected by qRT-PCR and western blot. **C, D** The transfection of miR-146a-5p mimic could not influence the expression of P27 detected by qRT-PCR and western blot. Data are mean±SD, n=3

**Supplemental Table**

**TABLE S1:** All PCR primer used in this research

| Primers and probes |  | sequence |
| --- | --- | --- |
| CircFAM114A2 | Forward  Reverse | 5’-GGGGCAAGTCCATACT-3’  5’-TTGGCTGGCTCACAG-3’ |
| FAM114A2 | Forward  Reverse | 5’-ACTGCTGTTCAGAGCACAGG-3’  5’-CCTTCTGCTATCACATCCATTGT-3’ |
| miR-222-3p (mimics)  miR-146a-5p (mimics)  U6  P27  P21  β-Actin  GADPH | Forward  Reverse  Stem-loop primer  Forward  Reverse  Stem-loop primer  Forward  Reverse  Forward  Reverse  Forward  Reverse  Forward  Reverse  Forward  Reverse | 5’-CGGCACGGGCCGAGGC-3’  5’-AGTGCAGGGTCCGAGGTATT-3’  5’-GTCGTATCCAGTGCAGGGTCCGAGGTATTCGCACTGGATACGACGCTGGT-3’  5’-GTTCTTCAGCTGGGATATCTCTGT-3’  5’-AGTGCAGGGTCCGAGGTATT-3’  5’-GTCGTATCCAGTGCAGGGTCCGAGGTATTCGCACTGGATACGACACGATG-3’  5’-AAAGCAAATCATCGGACGACC-3’  5’-GTACAACACATTGTTTCCTCGGA-3’  5’-AACGTGCGAGTGTCTAACGG-3’  5’-CCCTCTAGGGGTTTGTGATTCT-3’  5’-TGTCCGTCAGAACCCATGC-3’  5’-AAAGTCGAAGTTCCATCGCTC-3’  5’-CTCCATCCTGGCCTCGCTGT-3’  5’-GCTGTCACCTTCACCGTTCC-3’  5’-CAATGACCCCTTCATTGACC-3’  5’-GACAAGCTTCCCGTTCTCAG-3’ |
