# Supplementary figures and images for: CircFAM114A2 Promotes Cisplatin Sensitivity via miR-222-3p/P27 and miR-146a-5p/P21 Cascades in Urothelial Carcinoma
Source: Front Oncol. 2021 Oct 14;11:659166. doi: 10.3389/fonc.2021.659166 (PMC8551855; doi:10.3389/fonc.2021.659166)

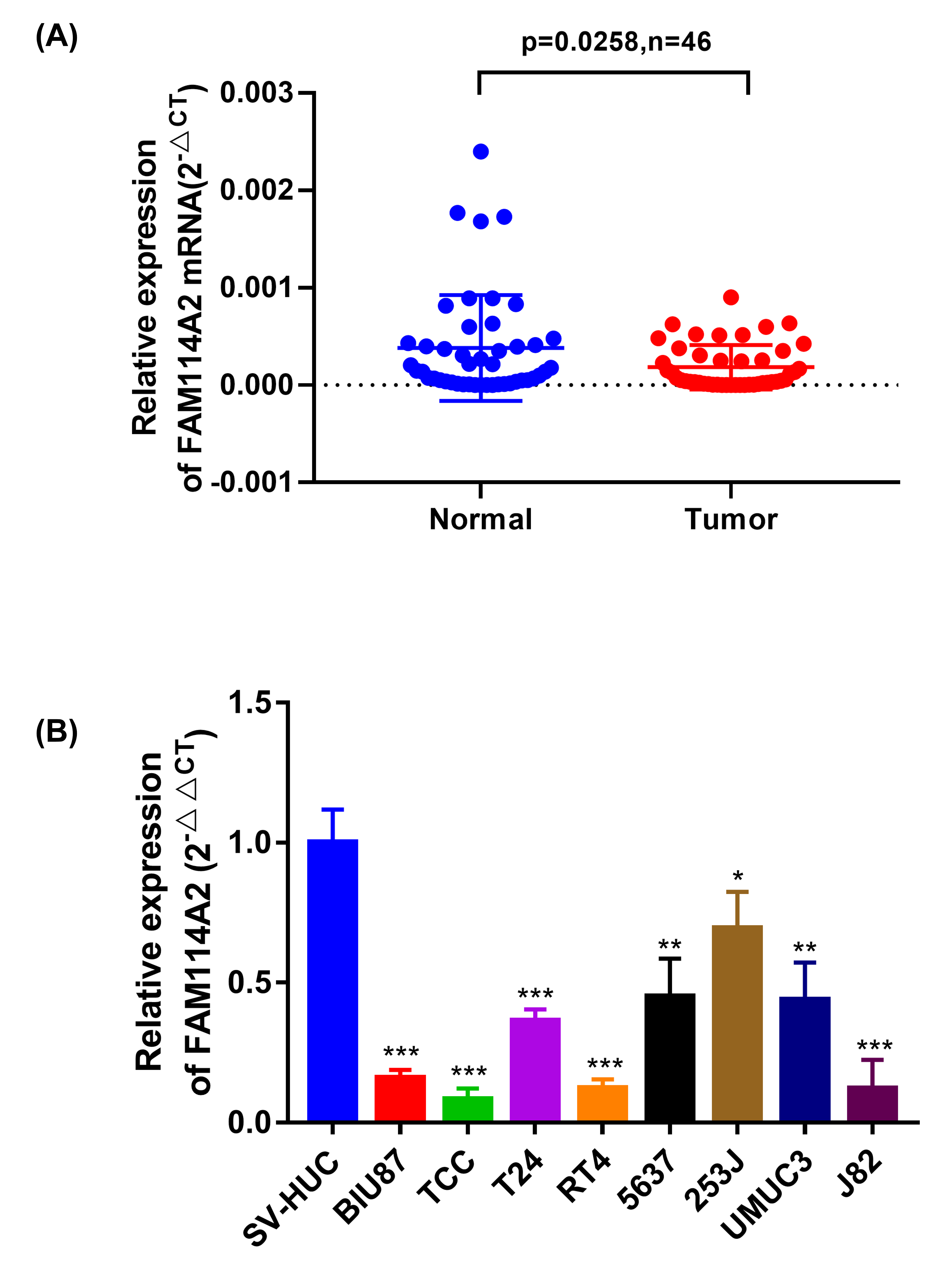

Supplement: Supplementary file 2 [file Image_1.png]

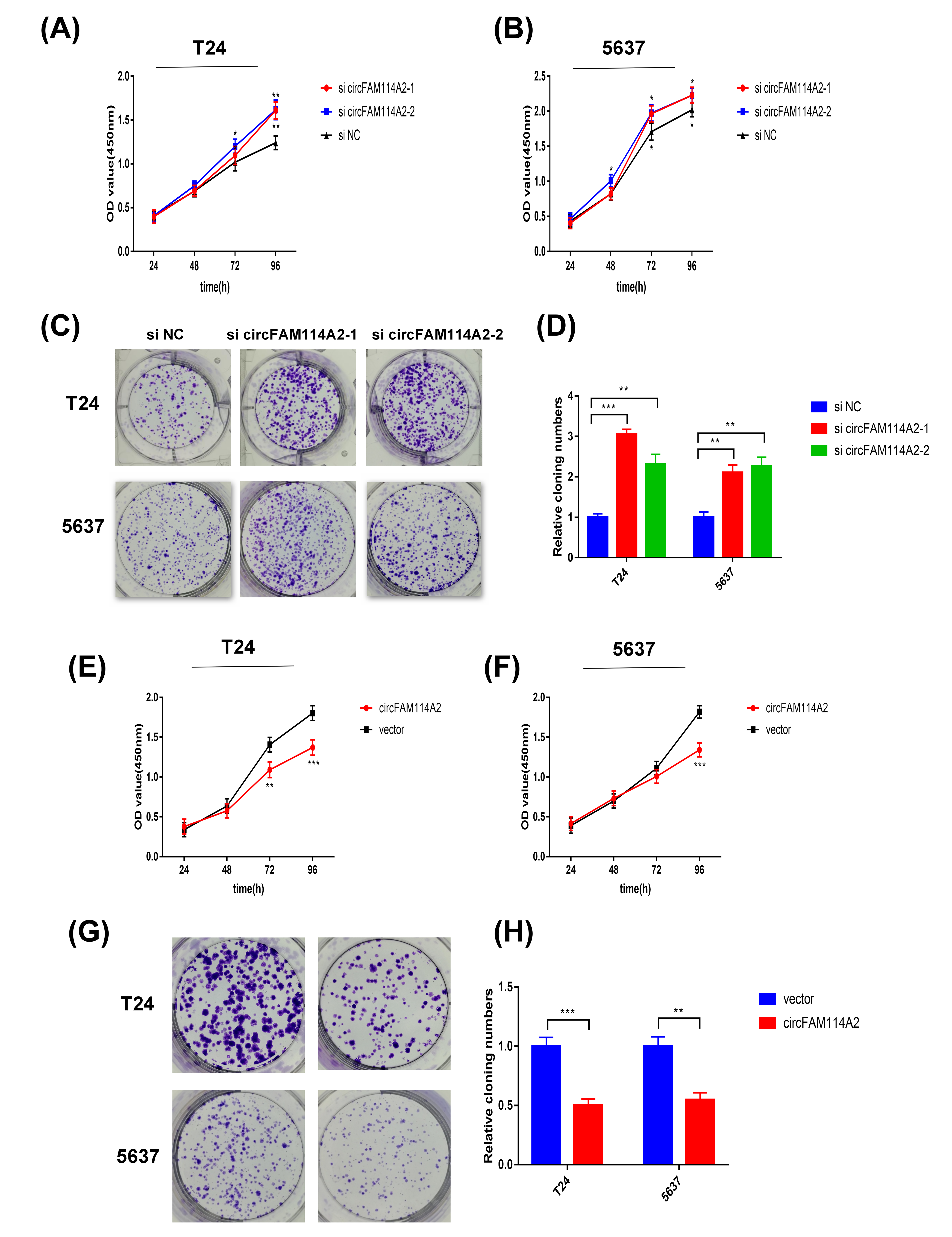

Supplement: Supplementary file 4 [file Image_3.png]

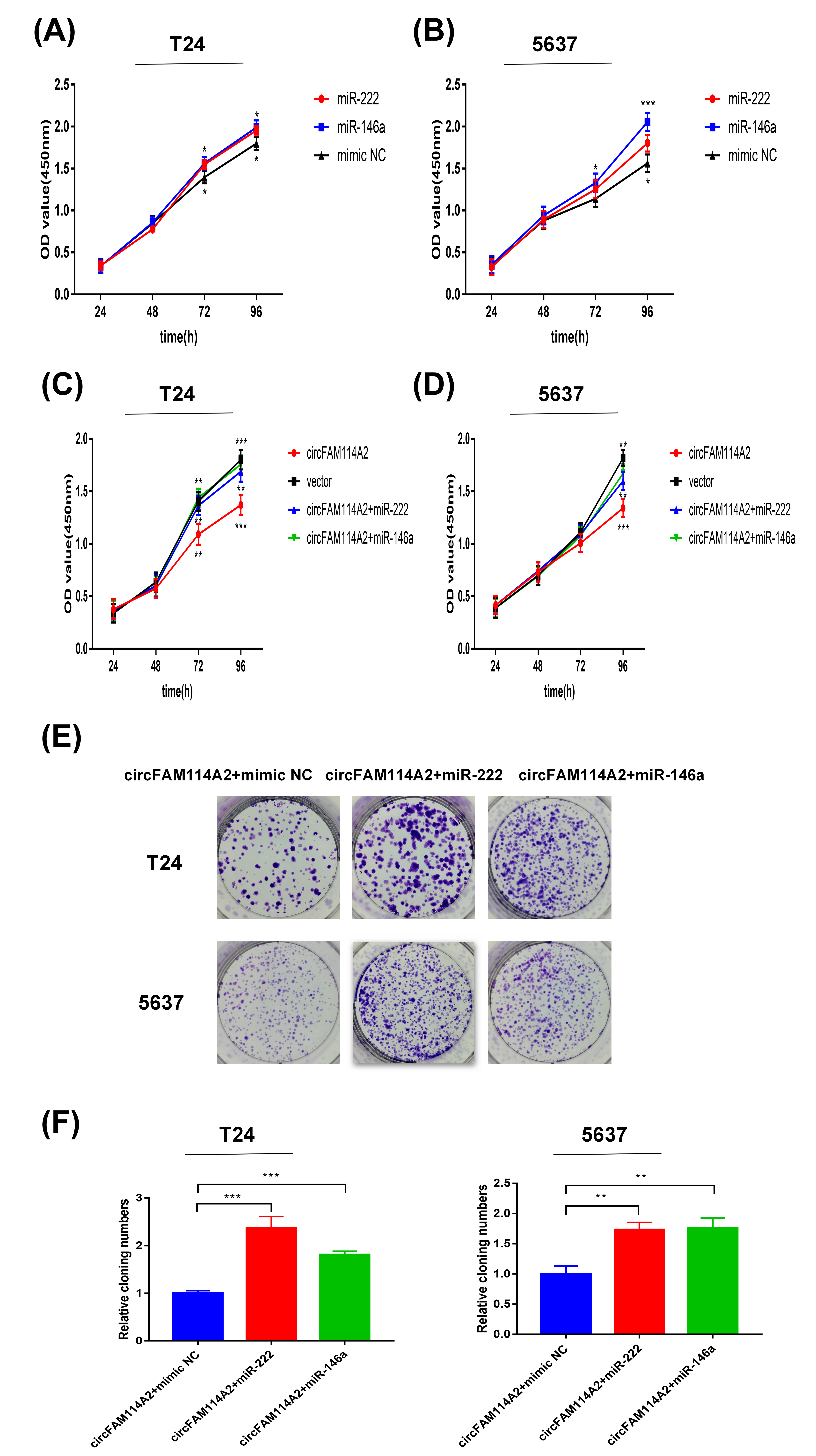

Supplement: Supplementary file 7 [file Image_6.png]

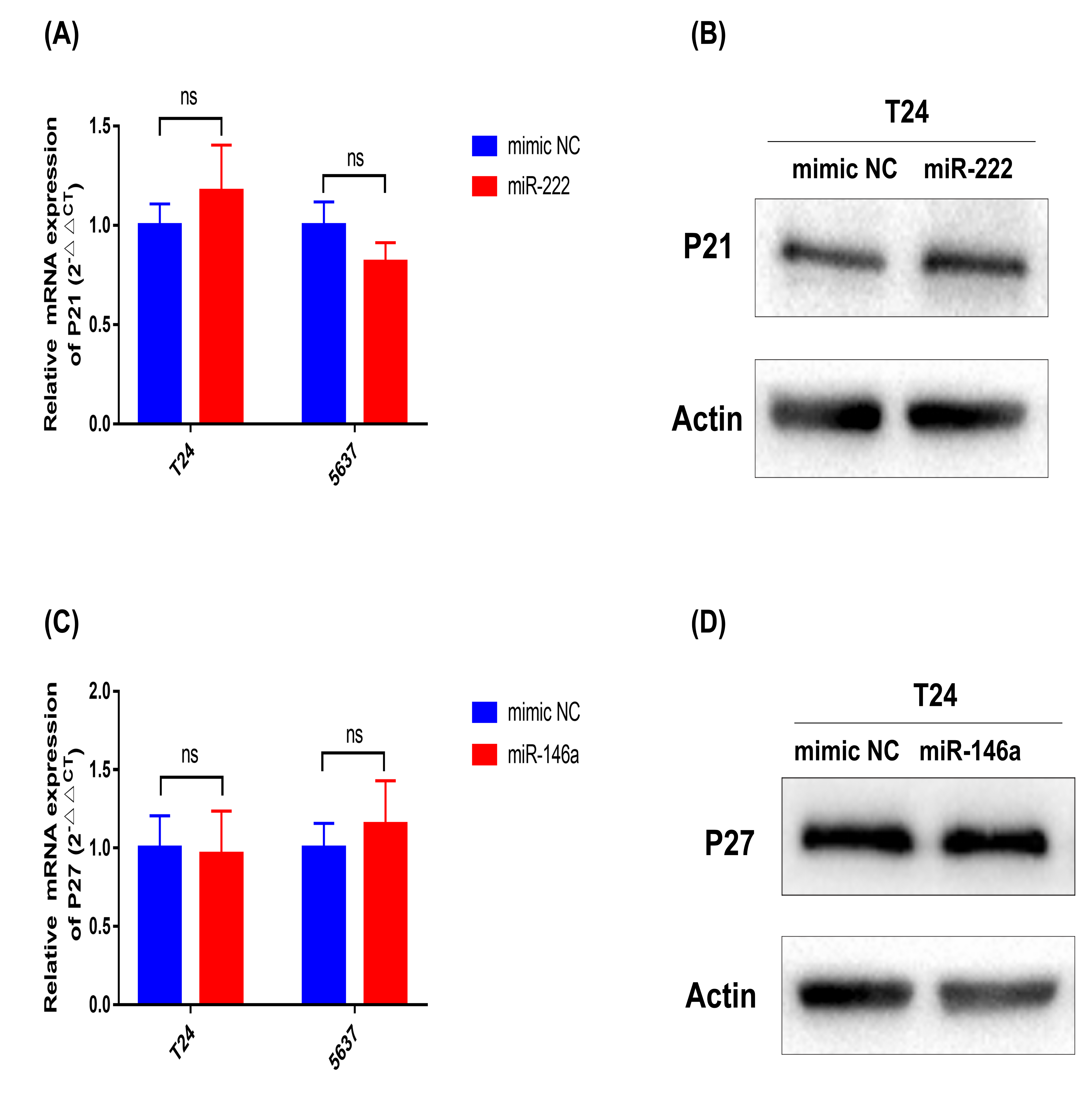

Supplement: Supplementary file 8 [file Image_7.png]
